# Mediterranean Diet Adherence and Its Association With Pain and Functional Status in Knee Osteoarthritis: A Cross-Sectional Study

**DOI:** 10.7759/cureus.110743

**Published:** 2026-06-12

**Authors:** Ghizlane Ghoullam, Imane El Binoune, Bouchra Amine, Ikram El Moubarik, Zeineb Alaoui Kadiri, Salma Zemrani, Samira Rostom, Rachid Bahiri

**Affiliations:** 1 Rheumatology, Ibn Sina Hospital Center, Faculty of Medicine and Pharmacy, Mohammed V University, Rabat, MAR

**Keywords:** functional impairment, knee osteoarthritis, medas score, mediterranean diet, pain

## Abstract

Background: Knee osteoarthritis (KOA) is a leading cause of chronic pain and disability worldwide, with metabolic and inflammatory mechanisms increasingly recognized in its pathophysiology. The Mediterranean diet (MD) has anti-inflammatory and cardiometabolic effects, but evidence regarding its association with pain and functional outcomes in KOA remains limited, particularly in North African populations.

Objective: The aim of our study was to assess the association between MD adherence and pain intensity in patients with primary KOA and to evaluate its relationship with functional impairment and clinical predictors of worse outcomes.

Methods: This single-center cross-sectional study included patients with primary KOA and was conducted at the Rheumatology Department A, El Ayachi Hospital, affiliated with the Ibn Sina University Hospital Center (CHU Ibn Sina), Rabat-Salé, Morocco. MD adherence was assessed using the Mediterranean Diet Adherence Screener (MEDAS) and classified into low (≤7) and high (>7) adherence groups. Pain was measured using the Visual Analog Scale (VAS) and functional impairment using the Lequesne index. Associations between dietary adherence and outcomes were analyzed using multivariate linear regression adjusted for key clinical and lifestyle confounders.

Results: A total of 103 patients were included (mean age 61.0 ± 9.97 years; 88.3% female). Higher MEDAS scores were independently associated with lower pain intensity (β = −0.947, p < 0.001) and better functional outcomes (Lequesne index: β = −1.967, p < 0.001). By contrast, higher BMI and advanced radiographic stages were significantly associated with increased pain and disability. Age, disease duration, and physical activity were not independently associated with outcomes.

Conclusion: Greater adherence to the MD is independently associated with reduced pain and improved functional status in patients with KOA. These findings support the potential role of dietary patterns as a complementary, non-pharmacological strategy in osteoarthritis (OA) management.

## Introduction

Knee osteoarthritis (KOA) is the most prevalent form of osteoarthritis (OA), represents a major cause of chronic pain and disability, and constitutes a substantial socioeconomic burden. It is estimated that more than 300 million people are affected globally, with prevalence increasing with age and obesity rates [1,2]. Among adults 60 years of age or older, the prevalence of symptomatic knee OA is approximately 10% in men and 13% in women [3]. 

OA has a multifactorial etiology and can be considered the product of an interplay between systemic and local factors [4]. Historically considered a "wear and tear" disease, OA is now recognized as a complex, multifactorial disorder involving mechanical, inflammatory, metabolic, and biomechanical mechanisms [5,6]. The risk factors for OA include age, gender, a history of trauma, excess weight and obesity, mechanical factors, and genetic predisposition [7]. In particular, metabolic syndrome and obesity have emerged as major contributors to both structural progression and pain amplification in KOA. Several studies show that patients with OA and metabolic syndrome have a higher incidence of inflammation and pain compared to those with OA without metabolic syndrome [8,9].

Since metabolic dysfunction is strongly associated with OA, lifestyle interventions are now a key part of its non-pharmacological management. The international guidelines for the management of OA include non-pharmacological strategies, pharmacological therapies, and surgical interventions. Among non-pharmacological approaches, physical activity and weight reduction are strongly recommended [10,11].

Among dietary patterns, the Mediterranean diet (MD) has gained considerable attention due to its well-documented anti-inflammatory and cardiometabolic benefits. The MD is characterized by high consumption of fruits, vegetables, legumes, whole grains, olive oil, fish, and low intake of red meat and processed foods. This dietary pattern is rich in antioxidants, polyphenols, and monounsaturated fatty acids. By contrast, high-fat diets have been associated with increased leptin levels in cartilage, accelerating OA progression, whereas diets rich in antioxidants, such as the MD, may help prevent cartilage degradation [12].

Mechanistically, the MD may influence OA through several pathways: reduction of systemic inflammation, improvement in lipid profile, modulation of gut microbiota, and indirect effects through weight control [13].

Despite increasing evidence of the anti-inflammatory and metabolic benefits of the MD, research specifically investigating its impact on pain severity and functional impairment in patients with KOA remains limited, particularly in North African populations.

Understanding whether adherence to the MD is associated with reduced pain and improved joint function in patients with KOA could provide a simple, low-cost adjunctive strategy in OA management.

The primary objective of this study was to assess the association between adherence to the MD and pain intensity in patients with primary KOA.

The secondary objectives were (1) to evaluate the association between MD adherence and functional impairment and (2) to identify clinical, metabolic, and lifestyle factors associated with higher pain levels and poorer functional outcomes.

## Materials and methods

Study design

This cross-sectional study was conducted in the Rheumatology Department of El Ayachi Hospital, affiliated with the Ibn Sina University Hospital Center (CHU Ibn Sina), Rabat-Salé, Morocco. Patients with primary KOA were consecutively recruited during routine outpatient consultations over a 12-month period, from January 2025 to January 2026. The study protocol was approved by the Ethics Committee for Biomedical Research at Mohammed V University, Faculty of Medicine and Pharmacy of Rabat (CERB 22-24). Written informed consent was obtained from all participants prior to inclusion.

Participants

Adults aged 18 years or older with a diagnosis of primary KOA based on clinical and radiographic criteria were included. Clinical diagnosis was established in the presence of mechanical knee pain and characteristic OA symptoms in the absence of inflammatory features. Radiographic diagnosis was confirmed by typical osteoarthritic changes, including osteophyte formation, joint space narrowing, subchondral sclerosis, and subchondral cysts. Radiographic severity was assessed using the Kellgren-Lawrence classification. Patients with secondary OA (post-traumatic or inflammatory origin), prior knee arthroplasty, recent dietary intervention or nutritional supplementation, intra-articular injection within the preceding three months, or use of non-steroidal anti-inflammatory drugs within seven days prior to inclusion were excluded to minimize potential confounding effects on pain, functional status, and dietary assessment.

Data collection

Sociodemographic characteristics, including age and sex, as well as clinical variables, such as disease duration and comorbidities, were recorded. Anthropometric measurements, including height, weight, and body mass index (BMI), were collected. BMI was categorized according to the World Health Organization criteria: normal weight (18.5-24.9 kg/m²), overweight (25-29.9 kg/m²), moderate obesity (30-39.9 kg/m²), and severe obesity (≥40 kg/m²). The anthropometric measurements were performed during routine clinical assessment using a standardized scale, with participants wearing light clothing. Measurements were recorded once.

Radiographic staging of KOA and the presence of OA at other sites were also documented.

Pain intensity was assessed using a Visual Analogue Scale (VAS) ranging from 0 to 10. Functional disability was evaluated using the Lequesne index, which assesses pain, walking distance, and activities of daily living, with severity classified as mild (Lequesne index ≤7), moderate (Lequesne index 8-10), or severe (Lequesne index ≥11) according to established cut-offs.

Physical activity was evaluated using the International Physical Activity Questionnaire (IPAQ) short form, a validated instrument assessing self-reported activity over the previous seven days. Results were expressed in MET-minutes per week and categorized into low (≤600 MET-min/week), moderate (600-3000 MET-min/week), and high (>3000 MET-min/week) activity levels. The IPAQ short form has been widely validated and used in epidemiological studies [14].

Adherence to the MD was assessed using the 14-item Mediterranean Diet Adherence Screener (MEDAS), originally developed and validated within the PREDIMED study and previously adapted and validated in Moroccan Arabic populations [15,16,17]. Permission to use the MEDAS questionnaire was obtained from the PREDIMED Study investigators. The MEDAS was interviewer-administered by a trained investigator in Moroccan Arabic or French according to participant preference. The MEDAS evaluates consumption of key food groups characteristic of the MD, including olive oil, fruits, vegetables, legumes, fish, nuts, red meat, and processed foods, as well as moderate alcohol intake. The total score ranges from 0 to 14, with higher scores indicating greater adherence. In this study, adherence was categorized based on a cut-off of 7, with scores above 7 indicating higher adherence according to previously described distributions in similar populations.

All clinical assessments were performed by a single trained investigator to ensure consistency and reduce measurement variability.

Statistical analysis 

All statistical analyses were performed using Jamovi software version 2.3.28. Continuous variables were expressed as mean ± standard deviation or median (interquartile range), depending on normality, while categorical variables were expressed as frequencies and percentages.

Comparisons between groups (low vs. high MD adherence) were performed using Student’s t-test or Mann-Whitney U test for continuous variables, and Chi-square or Fisher’s exact test for categorical variables.

Multivariate linear regression analyses were conducted to identify independent associations between MD adherence, pain intensity (VAS score, continuous), and functional impairment (Lequesne index).

Models were adjusted for potential confounders, including age, BMI, physical activity level, fatigue, comorbidities, and radiographic severity (Kellgren-Lawrence grade). Regression coefficients (B) with 95% confidence intervals were reported. Physical activity was assessed using the International Physical Activity Questionnaire (IPAQ) short form. IPAQ categories (low, moderate, or high activity) were described as part of baseline characteristics. For comparative analyses between the MD adherence groups, IPAQ was not included due to the low number of participants in the high activity category. However, IPAQ was retained in multivariate regression models as a potential confounding lifestyle factor. Assumptions of linear regression (normality, homoscedasticity, and multicollinearity) were verified prior to analysis.

No formal sample size calculation was performed. All eligible patients attending the outpatient rheumatology clinic during the study period were consecutively included until the predefined recruitment period ended. This design was chosen to ensure feasibility and reflect real-world clinical practice. A p-value <0.05 was considered statistically significant.

## Results

Study population characteristics

A total of 103 patients were included in the study. The mean age was 61.0 ± 9.97 years, and 91 (88.3%) were female. The mean BMI was 30.1 ± 4.82 kg/m², reflecting an overweight/obese population. Nearly half of the patients (51; 49.5%) had low physical activity levels, while only six (5.8%) had high activity. Most patients presented mild functional impairment according to the Lequesne index (65; 63.1%). Regarding treatments, 86 (83.5%) patients received rehabilitation, 42 (41.2%) used nonsteroidal anti-inflammatory drugs (NSAIDs), and 99 (96.1%) reported analgesic use (Table 1).

**Table 1:** Demographic, cardiometabolic, functional, and treatment characteristics of the study population (*) Data are expressed as mean ± standard deviation or median and interquartile range (IQR). (**) Data are expressed as number (percentage). BMI: body mass index; HDL: high-density lipoprotein; LDL: low-density lipoprotein; HbA1c: glycated hemoglobin; VAS: Visual Analog Scale; IPAQ: International Physical Activity Questionnaire; NSAIDs: non-steroidal anti-inflammatory drugs; MEDAS: Mediterranean Diet Adherence Screener

Variables	Patients (N= 103)
Age (years)*	61.0 ± 9.97
Female sex, n (%) *	91(88.3%)
Disease duration (years)*	4 (2-6)
BMI (kg/m²)*	30.1 ± 4.82
Mediterranean diet adherence* (%)	
Low adherence (MEDAS≤7)	42 (40.8%)
High adherence (MEDAS >7)	61 (59.2%)
Kellgren–Lawrence grade n (%)	
Grade I	14 (13.6%)
Grade II	55 (53.4%)
Grade III	25 (24.3%)
Grade IV	9 (8.7%)
BMI categories	
Normal weight**	17 (16.5%)
Overweight**	35 (34.0%)
Moderate obesity**	38 (36.9%)
Severe obesity**	13 (12.6%)
Cardiometabolic comorbidities	
Hypertension**	45 (43.7%)
Diabetes mellitus**	12 (11.7%)
Dyslipidemia**	24 (23.3%)
Cardiopathy**	8 (7.8%)
Lipid and Glucose Panel	
HDL (mmol/L)*	0.45 (0.38-0.55)
LDL (mmol/L)*	1.21 (0.95-1.6)
Total cholesterol (mmol/L)*	2.1 (1.9-2.4)
Triglycerides (mmol/L)*	1.19 (0.9-1.5)
HbA1c (%)	5.6 (5.27-5.83)
Functional status	
Lequesne index – mild (≤7)**	65 (63.1%)
Lequesne index – moderate (8-10)**	22 (21.4%)
Lequesne index – severe (≥11)**	16 (15.5%)
Pain and symptom scores (VAS)	
VAS pain*	4 (3-6)
VAS disability*	4 (3-5)
Physical activity	
IPAQ low (<600)**	51 (49.5%)
IPAQ moderate (600–3000)**	46 (44.7%)
IPAQ high (>3000)**	6 (5.8%)
Treatments	
NSAID use **	42 (41.2%)
Analgesic use **	99 (96.1%)
Rehabilitation **	86 (83.5%)

Comparison between MD adherence groups

Low adherence to the MD was associated with older age, longer disease duration, and higher BMI (all p < 0.01). Hypertension and dyslipidemia were significantly more frequent in this group. No significant differences were observed in metabolic laboratory parameters or in most comorbidities. Rehabilitation was more frequently reported in the low-adherence group (p = 0.008).

Patients with high MEDAS scores had significantly lower pain intensity and better functional status, with reduced VAS scores and lower Lequesne index values (all p < 0.001) (Table 2).

**Table 2:** Comparison of low and high Mediterranean diet adherence groups (*) Data are expressed as mean ± standard deviation or median and interquartile range (IQR). (**) Data are expressed as a number (percentage). Student’s t-test (t) was used for normally distributed variables, Mann–Whitney U test (U) for non-normally distributed variables, and chi-square test (χ²) for categorical variables. BMI: body mass index; HDL: high-density lipoprotein; LDL: low-density lipoprotein; HbA1c: glycated hemoglobin; VAS: Visual Analog Scale; IPAQ: International Physical Activity Questionnaire; NSAIDs: non-steroidal anti-inflammatory drugs; MEDAS: Mediterranean Diet Adherence Screener

Variables	Low-adherence MEDAS ≤7 (N = 42)	High-adherence MEDAS >7 (N = 61)	Test statistic	p-value
Age (years)*	66.5 ± 9.3	57.2 ± 8.6	t = 5.22	<0.001
Disease duration (years)*	6 (4-7)	3 (2-5)	U = 620	0.005
BMI (kg/m²)*	31.7 ± 5.78	29.1 ± 3.69	t = 2.88	<0.001
Cardiometabolic comorbidities				
Hypertension**	27 (64.2%)	18 (29.6%)	χ² = 12.2	<0.001
Diabetes mellitus**	7 (16.7%)	5 (8.2%)	χ² = 1.73	0.18
Dyslipidemia**	14 (33.4%)	10 (16.4%)	χ² = 3.99	0.04
Cardiopathy**	5 (11.9%)	3 (4.9%)	χ² = 1.63	0.21
Laboratory parameters				
HDL (mmol/L)*	0.49 (0.42-0.54)	0.45 (0.37-0.55)	U = 719	0.63
LDL (mmol/L)*	1.2 (0.92-1.6)	1.21 (0.98-1.6)	U = 752	0.7
Total cholesterol (mmol/L)*	2.2 (2.01-2.65)	1.98 (1.85-2.25)	U = 619	0.09
Triglycerides (mmol/L)*	1.23 (0.98-1.65)	1.1 (0.85-1.32)	U = 681	0.28
HbA1c (%)*	5.6 (5.37-6.02)	5.4 (5.2-5.73)	U = 717	0.06
Pain and functional outcomes				
VAS pain*	6 (5-7)	4 (3-4)	U = 159	<0.001
VAS disability*	6 (5-7)	3 (2-4)	U = 242	<0.001
Lequesne index*	9.9 ± 2.34	5.3 ± 1.52	t = 12	<0.001
Treatments				
NSAID use**	14 (33.4%)	28 (45.9%)	χ² = 2.54	0.11
Analgesic use**	41 (97.6%)	58 (95.1%)	χ² = 0.43	0.51
Rehabilitation**	40 (95.3%)	46 (75.5%)	χ² = 7.1	0.008

Independent predictors of pain intensity in KOA

In multivariate analysis, a higher MEDAS score was independently associated with lower pain intensity (β = −0.947, p < 0.001). By contrast, higher BMI (β = 0.047, p = 0.004) and higher radiographic stages (stage II: β = 0.711; stage III: β = 1.920; stage IV: β = 2.340; all p ≤ 0.003) were associated with increased pain. Age, disease duration, and IPAQ were not significant predictors (Table 3).

**Table 3:** Multivariate analysis of factors associated with pain intensity in knee osteoarthritis BMI: body mass index; IPAQ: International Physical Activity; MEDAS: Mediterranean Diet Adherence Screener

Predictor	β	95% CI	p-value
Age	0.009	-0.009-0.026	0.326
BMI	0.047	0.016-0.079	0.004
Disease duration	0.004	-0.036-0.044	0.841
Radiographic stage II vs. I	0.711	0.242-1.181	0.003
Radiographic stage III vs. I	1.920	1.294-2.545	<0.001
Radiographic stage IV vs. I	2.340	1.538-3.141	<0.001
MEDAS score (high vs. low)	−0.947	-1.354-−0.541	<0.001
Rehabilitation (no vs. yes)	−0.459	-0.873--0.045	0.030
IPAQ moderate vs. low	−0.100	-0.411-0.210	0.522
IPAQ high vs. low	−0.049	-0.715-0.617	0.884

Independent predictors of functional impairment (Lequesne index)

A higher MEDAS score was independently associated with a lower Lequesne index (β = -1.967, p < 0.001). Conversely, age (β = 0.053, p = 0.004), BMI (β = 0.085, p = 0.009), and radiographic severity were significant predictors of worse functional status. IPAQ level was not associated with functional impairment (Table 4).

**Table 4:** Multivariate linear regression analysis for factors associated with Lequesne index BMI: body mass index; IPAQ: International Physical Activity; MEDAS: Mediterranean Diet Adherence Screener

Predictor	β	95% CI	p-value
Age	0.053	0.018-0.088	0.004
BMI	0.085	0.022-0.148	0.009
Disease duration	0.062	-0.018-0.141	0.128
Radiographic stage II vs. I	1.477	0.567-2.387	0.002
Radiographic stage III vs. I	3.262	2.047-4.477	<0.001
Radiographic stage IV vs. I	4.524	2.955-6.093	<0.001
MEDAS score (high vs. low)	-1.967	-2.780--1.155	<0.001
IPAQ moderate vs. low	-0.120	-0.733-0.492	0.697
IPAQ high vs. low	0.868	-0.462-2.197	0.198

## Discussion

In this cross-sectional study of patients with KOA, higher adherence to the MD (MEDAS score) was independently associated with lower pain intensity and better functional status. By contrast, higher BMI and more advanced radiographic stages were consistently associated with worse pain and disability. Physical activity level and disease duration were not significantly associated with either pain or functional outcomes in multivariate analysis.

Our findings are consistent with previous studies suggesting a protective role of the MD on musculoskeletal pain and OA symptoms. Several observational studies have reported that adherence to a Mediterranean dietary pattern is associated with reduced inflammatory burden and lower pain severity in patients with OA and other chronic musculoskeletal conditions [18,19].

Our findings are supported by a randomized controlled trial conducted by Dyer et al., suggesting that Mediterranean-type dietary patterns may influence OA-related outcomes. Dyer et al. demonstrated in a 16-week randomized controlled trial that an MD intervention significantly reduced inflammatory markers, including IL-1α, and decreased serum cartilage oligomeric matrix protein (sCOMP), a biomarker of cartilage degradation in patients with OA [18]. These results may help explain the association observed in our study between higher MEDAS scores and lower pain intensity, suggesting that the MD may modulate pain through anti-inflammatory and chondroprotective mechanisms.

These findings are in line with a large prospective cohort study, including 4,330 participants followed over four years, which Veronese et al. showed that higher adherence to the MD was associated with a reduced risk of pain worsening (RR = 0.96; 95% CI: 0.91-0.999) and a lower risk of symptomatic KOA (RR = 0.91; 95% CI: 0.82-0.998) [19].

The potential mechanisms underlying these associations include reduction of systemic inflammation through decreased levels of pro-inflammatory cytokines such as IL-6 and TNF-α, improved oxidative stress profile, and modulation of adipokine activity, particularly leptin, which is known to contribute to cartilage degradation.

Several studies have demonstrated that synovial inflammation (synovitis) in OA is linked to more severe joint symptoms, accelerated cartilage degradation, reduced mobility, and higher radiographic severity scores [20]. Cytokines present in the synovial fluid promote cartilage matrix degradation by enhancing chondrocyte catabolic pathways while simultaneously suppressing their anabolic functions [21].

Beyond pain, our study demonstrated a significant association between MD adherence and better functional status as assessed by the Lequesne index. This finding is in line with previous studies reporting that healthier dietary patterns are associated with improved physical function and reduced disability in OA patients [22,23]. Diet quality may influence functional outcomes indirectly through weight control, reduced systemic inflammation, and improved cardiometabolic profile, all of which are known contributors to disability in KOA.

Interestingly, olive oil, a key component of the MD, has been shown to reduce pain and WOMAC scores and to improve functional outcomes, including the Health Assessment Questionnaire-Disability Index, in patients with OA [24].

Beyond structural severity, obesity represents a key determinant of OA symptomatology. In our study, higher BMI was independently associated with increased pain and worse functional impairment, in line with previous evidence highlighting the dual mechanical and metabolic role of excess weight in KOA.

This is consistent with interventional data showing that dietary modification may influence both body weight and symptom severity. In a randomized feeding trial, Sadeghi et al. demonstrated that an MD significantly reduced pain, morning stiffness, and improved physical function compared with a low-fat diet over 12 weeks. Importantly, the MD group showed greater reductions in pain during walking and standing, as well as a significant decrease in stiffness and weight parameters. These findings support the hypothesis that part of the beneficial effect of dietary patterns on OA symptoms may be mediated through metabolic factors, including weight reduction and improved systemic inflammatory status [23].

Furthermore, emerging evidence suggests that gut microbiota may represent an additional mechanistic pathway linking MD adherence and OA outcomes. A Mediterranean dietary pattern, rich in fiber, polyphenols, and unsaturated fatty acids, has been shown to increase gut microbial diversity and promote the abundance of beneficial bacterial taxa. This is associated with the production of short-chain fatty acids, particularly butyrate, which exert anti-inflammatory and immunomodulatory effects [25,26].

Interestingly, physical activity level and disease duration were not independently associated with pain or functional outcomes in our multivariate models. The lack of association with physical activity may be explained by limitations of self-reported questionnaires such as the IPAQ, which may not accurately capture real functional load or long-term activity patterns in OA patients.

Disease duration was not associated with symptom severity, supporting the concept that OA symptoms are more closely related to metabolic and structural factors rather than disease chronicity.

Strengths and limitations

This study has several strengths. First, it assessed the association between MD adherence and both pain and functional outcomes in KOA using validated and widely used instruments. Second, the study was conducted in a real-world clinical setting, enhancing its external validity and reflecting routine clinical practice in patients with KOA. Finally, the inclusion of both clinical and metabolic variables provides a comprehensive overview of disease determinants.

However, several limitations should be acknowledged. The cross-sectional design of the study does not allow causal inference between MD adherence and OA outcomes. Reverse causality cannot be excluded, as patients with more severe symptoms may have modified their dietary habits.

Moreover, the study was conducted in a single center with a relatively modest sample size, which may limit the generalizability of the findings. The lack of longitudinal follow-up prevents assessment of the long-term impact of dietary adherence on disease progression.

Although both the MEDAS and IPAQ questionnaires were interviewer-administered, information bias cannot be completely excluded, and recall bias may still be present due to participants’ reliance on memory of dietary intake and physical activity patterns.

Furthermore, radiographic assessment of KOA, although based on standard criteria, may be subject to inter-observer variability in grading.

Clinical implications

From a clinical perspective, our findings suggest that dietary counseling could be considered as part of a multidisciplinary management strategy for KOA. However, given the cross-sectional design of this study, causal relationships cannot be established. Future longitudinal and interventional studies are needed to determine whether improving MD adherence influences pain trajectories and functional outcomes.

## Conclusions

This study highlights a significant association between adherence to the MD and both pain and functional outcomes in KOA. Beyond its clinical associations, these findings reinforce the relevance of dietary patterns as part of the broader metabolic and inflammatory context of OA.

Given the multifactorial nature of KOA, integrating nutritional approaches, such as the MD, may represent a useful adjunct to conventional management strategies. Further longitudinal and interventional studies are warranted to better define its role in disease modulation and symptom control.
